# Inhibition of vascular adhesion protein 1 protects dopamine neurons from the effects of acute inflammation and restores habit learning in the striatum

**DOI:** 10.1186/s12974-021-02288-8

**Published:** 2021-10-15

**Authors:** Serena Becchi, Alberto Buson, Bernard W. Balleine

**Affiliations:** 1 Decision Neuroscience Lab, School of Psychology, UNSW Sydney, Randwick, NSW 2052 Australia; 2 Aegros Pharmaceuticals, Sydney, Australia

**Keywords:** VAP-1, Dopamine neurons, Inflammation, Dorsolateral striatum, Blood–brain barrier, Habit learning

## Abstract

**Background:**

Changes in dopaminergic neural function can be induced by an acute inflammatory state that, by altering the integrity of the neurovasculature, induces neuronal stress, cell death and causes functional deficits. Effectively blocking these effects of inflammation could, therefore, reduce both neuronal and functional decline. To test this hypothesis, we inhibited vascular adhesion protein 1 (VAP-1), a membrane-bound protein expressed on the endothelial cell surface, that mediates leukocyte extravasation and induces oxidative stress.

**Method:**

We induced dopaminergic neuronal loss by infusing lipopolysaccharide (LPS) directly into the substantia nigra (SN) in rats and administered the VAP-1 inhibitor, PXS-4681A, daily.

**Results:**

LPS produced: an acute inflammatory response, the loss of dopaminergic neurons in the SN, reduced the dopaminergic projection to SN target regions, particularly the dorsolateral striatum (DLS), and a deficit in habit learning, a key function of the DLS. In an attempt to protect SN neurons from this inflammatory response we found that VAP-1 inhibition not only reduced neutrophil infiltration in the SN and striatum, but also reduced the associated striatal microglia and astrocyte response. We found VAP-1 inhibition protected dopamine neurons in the SN, their projections to the striatum and promoted the functional recovery of habit learning. Thus, we reversed the loss of habitual actions, a function usually dependent on dopamine release in DLS and sensitive to striatal dysfunction.

**Conclusions:**

We establish, therefore, that VAP-1 inhibition has an anti-inflammatory profile that may be beneficial in the treatment of dopamine neuron dysfunction caused by an acute inflammatory state in the brain.

**Supplementary Information:**

The online version contains supplementary material available at 10.1186/s12974-021-02288-8.

## Background

Many acute and chronic neurodegenerative conditions are accompanied by an inflammatory state in the brain and yet how such changes induce neuronal effects is currently unclear [1, 2]. One important factor is the influence of brain inflammation on the integrity of the neurovasculature generally and the blood–brain barrier (BBB) in particular [3, 4]. An intact BBB is necessary for tissue homeostasis and to avoid collateral tissue damage whereas a dysfunctional BBB has been linked to various neurological disorders [5] and neurodegenerative diseases [6]. Under inflammatory conditions, the endothelium increases the expression and/or function of adhesion molecules and such endothelial activation has been described in both acute dysfunction [7] and in chronic neurodegenerative diseases such as Alzheimer's disease [8–10] and Parkinson’s disease [11–13] in humans, and in animal models of Parkinson’s disease [14–16].

Adhesion proteins control leukocyte migration from the blood stream to the tissue and each step of this migration event is regulated by specific adhesion molecules both on leukocytes and endothelial cells, which increase during inflammation [17]. Vascular adhesion protein 1 (VAP-1) is amongst these adhesion proteins. It is expressed on endothelial cells and binds leukocytes through its semicarbazide-sensitive amine oxidase activity (SSAO) [18–21]. It is key for neutrophil extravasation in vivo and, when the function of VAP-1 is inhibited, the net effect is the impairment of neutrophil migration into areas of inflammation [22].

As an enzyme, VAP-1 metabolizes primary amines, generating the corresponding aldehydes, hydrogen peroxide (H_2_O_2_) and ammonia, which are able to induce cellular damage when and where they are overproduced [23]. Under normal conditions VAP-1 is mainly intracellular, but under inflammatory stimulation it is expressed at the cell membrane of activated endothelial cells and is shed into the blood stream, increasing VAP-1 levels in the blood that can serve as a biomarker for inflammation [24]. VAP-1 expression is also altered in a number of human neuropathologies and animal models [25–32], common features of which are gliosis and leukocyte infiltration [11, 33] as well as the microvascular proliferation [12] that can precede and worsen vascular damage and neuronal loss.

The adhesive function of VAP-1 can be inhibited either by monoclonal antibodies or by small-molecule SSAO inhibitors [34] and, indeed, we have recently shown that the selective VAP-1 inhibitor, PXS-4681A [35], reverses the effects of systemic or ICV lipopolysaccharide (LPS) on microglia activation and neutrophil infiltration in the substantia nigra (SN) and striatum [36]. Here, we infused LPS into the SN to induce an acute inflammatory state sufficient to deplete dopaminergic (DA) neurons [37, 38] and attempted to block this effect with a VAP-1 inhibitor and so rescue DA neuronal loss, the loss of the DA projection in the striatum and associated functional deficits particularly in habit learning, a critical psychological function commonly lost in both acute and chronic conditions that interfere with neural function [39].

## Materials and methods

### Animals

Hooded Wistar male rats between 300 and 400 g obtained from Adelaide Laboratory Animal Services (University of Adelaide, Australia) were used in these experiments. Animals were housed in groups of up to 3–4 per cage in a 12/12 h light/dark cycle (7:00AM to 7:00PM) with full access to food and water and environmental enrichment. Throughout the behavioural experiment, rats were maintained at ∼85% of their free-feeding body weight by restricting their food intake. All animals were humanely killed via injection of pentobarbitone sodium in compliance with the ARRIVE guidelines [40–42].

### Drugs and drug treatments

Unless otherwise specified, chemicals reagents and solvents were purchased from Sigma Aldrich (St. Louis, MO). The VAP-1 inhibitor PXS-4681A, [(Z)-4-(2-(aminomethyl)-3fluoroallyloxy)benzenesulfonamide hydrochloride], was synthesized by Pharmaxis Ltd [35].

### Intranigral LPS lesion

Before the start of surgery, the animals received injections of antibiotic, Benacillin (Ilium) and the local anaesthetic Bupivacaine (Hospira) injected subcutaneously (sc) at the surgical site. Rats were anaesthetized with isoflurane (5% for induction and 2–3% for maintenance) and positioned in a stereotaxic frame (Stoelting, Wood Dale, IL, USA). An incision was made to expose the scalp and the incisor bar was adjusted to align bregma and lambda on the same horizontal plane.

#### Unilateral infusions

For all rats, holes were drilled into the skull at the following coordinates: − 5.3 mm posterior; ± 2.0 mm lateral; − 8.0 mm ventral to bregma [43]. Each unilateral injection of LPS from Escherichia coli (O55:B5) (2 µl of 1.5 mg/ml solution of LPS) into the SN was conducted using a glass capillary filled onto a Nanoject II (Drummond Scientific) mounted on the stereotaxic frame in order to minimize the mechanical damage of the needle to the tissue. Once in position, LPS was delivered at an approximate rate of 100 nL/min, plus an additional 10 min where the needle was left in situ to avoid reflux along the injection track. Warm saline (5 ml) was administered at the end of the surgical procedure.

#### Bilateral infusions

The procedures were identical as described for unilateral infusions. LPS was infused into the lateral SN at the following coordinates: − 5.3 mm posterior; ± 2.7 mm lateral; − 7.4 mm ventral to bregma [43], whereas a saline control group received bilateral injections of sterile saline into the lateral SN.

#### PXS-4681A treatment

1 h before and 5 h after LPS infusions, animals received 2 mg/kg of PXS-4681A dissolved in phosphate buffer saline (PBS), ip. Once a day injection was continued for the remainder of the experiment.

### Immunohistochemical procedures

At day 16 the animals were killed with pentobarbital sodium and transcardially perfused with 400 ml PBS at 4 °C followed by 400 ml of 4% paraformaldehyde (PFA) solution at 4 °C. Brains were fixed in the same fixative before being sectioned with a Leica VT 1000S Vibratome (Leica Microsystem). Coronal sections of 30 µm containing the SN or the striatum were washed 3 × 0.1 M at pH 7.4 PBS for 10 min.

#### Dopaminergic neurons

DA neurons were stained with mouse anti-tyrosine hydroxylase (TH) antibody in both SN and striatum and visualized using nickel-enhanced 3,3'-diaminobenzidine (DAB). After washing the sections in PBS 3 × 10 min, they were quenched for 10 min in 10% methanol and 3% H_2_O_2_. Sections were then rinsed 3 × 10 min in PBS and blocked in blocking solution PBS containing 5% normal serum and 0.25% Triton-X-100. The primary antibody was incubated in blocking solution overnight at 4 °C. Sections were washed and incubated in biotin-SP AffiniPure donkey anti-mouse IgG (Jackson ImmunoResearch, USA) for 2 h, they were then incubated with ABC (Avidin–Biotin Complex) Kit (VectorLabs, Australia) for 1 h. DAB development was conducted adding glucose oxidase in 0.1 M acetate buffer pH 6.0. Sections were mounted and coverslipped with Entellan® (Merk, Australia).

Cell count in the SN was performed live using an upright microscope (Olympus BX50, Japan) under a 10 × Zeiss LSM 7110 CLSM (Carl Zeiss, Germany) lens. Boundaries of the regions of interest were created using a 10 × 10 grid reticule (1 × 1 mm) located in the right eyepiece of the microscope. One in every six sections between bregma − 4.80 mm and − 6.12 mm [43] was counted manually. Values across sections were averaged for each animal. The operator was blind to treatment condition using a random renumbering of the samples before the beginning of data acquisition.

For the striatum, one in every six sections between bregma + 1.9 mm and 0.00 mm [43] was quantified with Open Source ImageJ software (MacBiophotonics upgrade version 1.43u, Wayne Rasband, National Institutes of Health, Bethesda, MD). Images of dorsal striatum were acquired with a 20 × Zeiss LSM CLSM lens using a confocal laser scanning microscope (FV1000, Olympus, Japan) in both ipsilateral and contralateral hemispheres. In each hemisphere, two different ROIs were defined: ROI 1 comprised the grey matter of the dorsal striatum and ROI 2 comprised the fibre bundles with no TH staining and was used as background correction. The mean grey value of TH staining was collected for each ROIs by ImageJ software, and expressed as ROI 1 – ROI 2. Again, the operator was blind to the groups using the random renumbering of samples before the beginning of the data acquisition by a different experimenter blind to the treatment condition, and the group assignment was not known until all analyses were complete. One grey value was obtained per animal.

Subjects with an inaccurate injection site were excluded from the statistical analysis during post-mortem assessment. Correct injection placements were considered when the needle track was found between − 5.10 and − 5.40 mm posterior to bregma [43] and touching the *pars compacta*.

### Immunofluorescence procedure and quantification

Unless specified, immunofluorescence staining was conducted as follows: after washing the sections in Tris-buffered saline (TBS) 3 × 10 min, they were left in the blocking solution for 1 h, followed by incubation in primary antibodies dissolved in blocking buffer overnight at 4 °C. After washing in TBS 3 × 10 min, they were incubated in secondary antibodies dissolved in blocking solution for 2 h. Sections were then rinsed in TBS 3 × 10 min, mounted and coverslipped with Vectashield (VectorLab, Australia). Images were acquired with a 20 × Zeiss LSM CLSM lens using a confocal laser scanning microscope (FV1000, Olympus) and intensity of the staining was quantified as mean grey value with Open Source ImageJ software. Refer to Table 1 for dilutions and product numbers of antibodies.

**Table 1:** List of antibodies (Key resources table)

Reagent or Resource	Source	Identifier
Antibodies		
Rabbit anti-glial fibrillary acidic protein	Abcam (1:1000)	AB7260
Rabbit anti-interleukin 1 beta	Santa Cruz	SC-7884
Rat anti-interleukin 10	Abcam (1:500)	AB33471
Goat anti-ionized calcium binding adapter molecule	Abcam (1:2000)	AB5076
Rabbit anti-ionized calcium binding adapter molecule	Wako (1:1000)	019–19741
Rabbit anti-myeloperoxidase	Abcam (1:300)	AB45977
Mouse anti-rat endothelial cell antigen	Serotec (1:300)	MCA970R
Mouse anti-tyrosine hydroxylase	Millipore (1:10,000)	AB1542
Rabbit anti-tumour necrosis factor alpha	Abcam (1:500)	AB6671
Rabbit anti-vascular adhesion protein 1	Abcam (1:300)	AB42885
Donkey anti-rat IgG Alexa Fluor® 594	Invitrogen (1:1000)	A21209
Donkey anti-rabbit IgG Alexa Fluor® 488	Invitrogen (1:1000)	A21206
Donkey anti-mouse IgG Alexa Fluor® 405	Invitrogen (1:1000)	A48257
Donkey anti-goat IgG Alexa Fluor® 546	Invitrogen (1:1000)	A11056
NeuroTrace™ 640/660 Nissl	Thermofisher (1:1000)	N21483
Donkey anti-goat IgG Alexa Fluor® 594	Invitrogen (1:1000)	A11057
Donkey anti-mouse IgG Alexa Fluor® 546	Invitrogen (1:1000)	A10036
Biotin-SP AffiniPure donkey anti-mouse IgG	Jackson ImmunoResearch (1:400)	715-065-150
Mouse anti-major histocompatibility complex II	Abcam (1:500)	AB23990
Mouse anti-postsynaptic density protein 95	Invitrogen (1:500)	MA1-045

Immunofluorescence on striatal sections was performed on four to six sections chosen every 0.35 mm from bregma + 1.90 to 0.12 mm [43] and each specific staining was quantified in an area of 635.9 µm (h) × 635.9 µm (w) in both hemispheres using ImageJ software.

Myeloperoxidase (MPO) staining was conducted slightly differently: samples were boiled for 6 min in 10 mM citrate buffer at pH 6.0 before immunostaining procedure. MPO antibody was incubated overnight with ionized calcium binding adaptor molecule (Iba)-1 and rat endothelial cell antibody (RECA)-1, and the combination of the three was used to identify neutrophils and their location relative to the blood vessels [36]. A combination of secondary antibodies (donkey anti-mouse IgG Alexa Fluor® 405, donkey anti-rabbit IgG Alexa Fluor® 488 and donkey anti-goat IgG Alexa Fluor® 546) and NeuroTrace™ 640/660 Nissl was used to visualize the staining. In the SN, images were acquired with 10 × Zeiss LSM 7110 CLSM (Carl Zeiss, Germany) lens at high resolution (4800 × 4800 pixels per image) and neutrophil cells were manually counted using Image J cell count. MPO-positive cells were counted in three sections between bregma − 4.80 mm and − 5.70 mm [43] that were 0.3 mm equidistant from the injection site. In the striatum, four sections between bregma + 1.90 and 0.12 mm [43] were counted. One value per animal per hemisphere and area was obtained.

Microglia cells were quantified as Iba1-positive cell number and Iba1-positive area with Image J. Cell count was performed with Image J cell counter plugin while for Iba-1-positive area a fix thresholding was applied to each image and measured.

Double staining for mouse anti-major histocompatibility complex (MHC) II and goat anti-Iba1 was carried as follow. The fluorescence analysis was performed so that a ROI comprised the microglia defined by Iba1-positive area. Mean grey intensity values for each marker were obtained quantifying the intensity of the staining of MHC II within the microglia-defined ROI. A single value of mean grey value was obtain in each hemisphere for each marker for each rat. All double stainings were visualized with Alexa Fluor® 488 and Alexa Fluor® 594 or Alexa Fluor 546® conjugated secondary antibodies.

Rabbit anti-glial fibrillary acidic protein (GFAP) was used to label astrocytes and quantified as GFAP-positive area in each image. GFAP-positive area was identified using a fix thresholding parameter using Image J. In the SN, the GFAP-positive area was quantified using an Olympus VS-120 slide scanner. Three ROIs were created in the LPS side and in the contralateral hemisphere in six coronal sections between bregma − 4.80 mm and − 6.12 mm [43], every 0.25 mm, covering the SN area. The average of intensity of staining was calculated with image J from each ROI and averaged per hemisphere and per animal.

VAP-1 expression was revealed with rabbit anti-VAP-1 and co-stained with anti-RECA-1 and revealed with donkey anti-rabbit IgG Alexa Fluor® 488 and donkey anti-mouse IgG Alexa Fluor® 546 secondary antibodies. NeuroTrace™ 640/660 deep red fluorescent Nissl was incubated with secondary antibodies for 1 h at room temperature.

Post-synaptic puncta were identified with mouse anti-post synaptic density (PSD)-95 and visualized with donkey anti-mouse Alexa Fluor® 546. Images of PSD-95 staining were taken with 60 × Zeiss LSM 7110 CLSM (Carl Zeiss, Germany), zoomed 2×. The intensity of PSD-95 staining was quantified in images representing an area of 35 µm (h) × 35 µm (w) in four sections from bregma + 1.90 and 0.12 mm [43], chosen every 0.45 mm in the dorsal striatum. A threshold was calculated for each image using Image J software and a mask was created. The values of PSD-95-positive staining as mean grey value and positive area were quantified in each image. Values of four sections were averaged for each hemisphere for each rat and treated as a single *n*.

#### Blood–brain barrier leakage

Breakdown of the blood–brain barrier (BBB) was assessed by employing a one-step immunohistochemical detection of IgG as in Tomás-Carmadiel et al. [44] and Schmidt-Kastner et al*.* [45]. Briefly, seven sections between bregma -4.80 mm and -6.12 mm [43] were incubated for 2 h in donkey anti-rat IgG Alexa Fluor® 594 (Invitrogen; 1:1000 in PBS containing 1% bovine serum albumin and 0.2% Triton-X-100) and then double-stained with rabbit anti-GFAP, followed by donkey anti-rabbit IgG Alexa Fluor® 488. Visualization of GFAP and IgG immunoreactivity was detected under 4 × Zeiss LSM 7110 CLSM (Carl Zeiss, Germany) and Image J was used to calculate the IgG-positive area per section.

### Rotational behavior

At day 14 animals were tested for spontaneous forelimb akinesia using a cylinder test and for rotational behaviour induced by apomorphine. The cylinder test assesses a rat's ability to use each forelimb to support its body against the wall of a cylindrical enclosure. We performed this test following the procedure reported by Schallert and Tillerson [46]. Briefly, rats were put individually in a glass cylinder (20 cm diameter, 30 cm height) and video recorded for 5 min. No habituation to the cylinder prior to filming was allowed. The test was performed between 10.00 and 14.00 h. Two mirrors were placed to the sides of the cylinder at an angle that enable the recording of forelimb movements even when the animal was turned away from the camera. Scoring was conducted by an experimenter blind to the experimental treatment using VLC software with slow-motion and clear stop-frame capabilities. The behaviour was scored for independent use of the left or right forelimb to contact the cylinder wall during a full rear to initiate a weight-shifting movement or to regain centre of gravity while moving laterally in a vertical posture [46].

Apomorphine-induced rotational behaviour was assessed the day after the cylinder test. Each rat was placed in a circular arena (30 cm diameter) for 5 min before receiving an ip injection of 0.5 mg/kg of apomorphine hydrochloride dissolved in 0.02% ascorbic acid and saline. Rotational behaviour was recorded for 30 min after apomorphine injection and scored later by an experimenter blind to treatment condition [47].

### Instrumental training and testing

#### Apparatus

All behavioural procedures were performed in 16 identical Med Associates (USA) operant chambers enclosed in sound- and light-attenuating shells. Each chamber was equipped with a pump that was fitted with a syringe that delivered 20% sucrose solution (0.1 ml) into a recessed food magazine. An infrared photobeam that crossed the magazine allowed for the detection of magazine head entries. Each chamber contained two retractable levers to the right and left of the magazine and a 3 W 24 V house light mounted on the top of the wall opposite the magazine provided illumination. Two microcomputers running on the Med-PC program (Med Associates) controlled experimental events and recorded lever presses and magazine entries.

#### Lever-press training for habit

Following 4 days of food deprivation, rats were given two sessions of magazine training. Sucrose solution was delivered at random 60 s intervals for 30 outcomes per session. Animals then received 8 days of instrumental training (two sessions per day) to press a single lever for sucrose solution delivery. Right and left levers were counterbalanced across animals. Rats initially received three sessions in which the sucrose was delivered on a continuous reinforcement schedule and then four sessions in which it was delivered on a random interval schedule of 15 s (RI-15), four sessions on a RI-30 schedule, and four sessions on a RI-60 schedule. Each session commenced with the insertion of the lever; sessions ended when 30 reinforcers were earned or after 60 min, whichever came first. All groups received the same total number reinforcers.

#### Outcome devaluation

The day after the last session of training, the sucrose solution was devalued using conditioned taste aversion. All rats were given ad libitum access to sucrose solution for 30 min each day for 3 consecutive days in clear plastic tubs with bottles filled with sucrose solution attached. On each day, half of each lesion group received an intraperitoneal injection of lithium chloride (0.15 M LiCl, 10 ml/kg) and were placed back in their home cages, whereas the other half received saline injections (10 ml/kg). During outcome devaluation no PXS-4681A was administered to the rats with the aim to minimize any possible secondary effect of the drug on devaluation. The amount of sucrose solution consumed each day was also measured.

#### Extinction test

Over the 2 days following the last day of outcome devaluation, all rats received two extinction tests. The tests began with the insertion of the same lever used during training and ended with the retraction of the lever after 10 min. Lever presses and magazine entries were recorded, but no sucrose reinforcers were delivered.

#### Lever-press training for goal-directed learning

After conditioned taste aversion devaluation and tests, a sub-cohort of rats was trained to receive two different types of outcome, purified pellets and grain pellets. One outcome was earned for pressing the left lever and the other for pressing the right lever. The identity of the lever–outcome relationships was counterbalanced across subjects.

Twenty outcomes on each lever were delivered per session. Rats received 10 days of instrumental training, during which time the left and right lever press responses were trained with the 2 different outcomes in separate sessions each day. The interval between the training sessions for each lever press action was 10 min. The order of the lever presentations was alternated and counterbalanced across rats and days. The rats received 2 days in which lever pressing was continuously reinforced (CRF); then, the probability of the outcome given a response was gradually shifted over days using increasing random ratio (RR) schedules; a RR5 schedule (probability of receiving an outcome given a response = 0.2) and a RR10 (probability of receiving an outcome given a response = 0.1). Rats received 3 RR5 sessions followed by 5 RR10 sessions.

#### Specific satiety outcome devaluation

Twenty-four hours after the final instrumental training session, rats were given an outcome devaluation test. In this test, rats received ad libitum access to 1 of the 2 outcomes for 1 h in clear plastic feeding cages. Half of the rats in each response-outcome assignment received grain pellets and the remaining rats received purified pellets. Immediately after devaluation, rats were given a 10-min choice extinction test in which both levers were available but no outcome was delivered. Testing in extinction ensured that the rats had to recall both the current action–outcome contingencies and the current values of the two instrumental outcomes to choose appropriately. The following day all rats were given a second test with the other outcome devalued; that is, rats that were pre-fed on grain pellets for the first devaluation test were now pre-fed with purified pellets and vice versa.

### Locomotor assessment with rotarod and grip strength meter

At the end of instrumental testing, motor balance and coordination were evaluated with a rotarod (Ugo Basile, Italy). Rats were trained on the rotating rod at a fixed speed of 4 rpm for 3 trials until they stayed on the rod for at least 2 min. On the following days, the speed increased from 4 to 50 rpm within 5 min. Each animal had three trials of testing on two separate days with a resting period of at least 2 min between each trial. The time the animal spent on the rotarod was measured as latency to fall and the average of the six trials was considered per rat.

Forelimb grip strength was measured after the rotarod test as maximum tensile force using a rat grip strength meter (Columbus Instruments, Columbus, USA) with a sensor range of 0–5,000 g, and accuracy of 0.15%. Ten trials per rat were recorded and the average of the highest 5 pulls of the grip strength meter was recorded for each animal.

### Statistical analysis

The data are expressed as mean ± SEM. For statistical significance, experiments with two groups were analysed using two-tailed *t*-tests (unpaired). Experiments with more than two groups were analysed using one-way or two-way ANOVA or repeated measures two-way ANOVA, as appropriate, followed by Sidak’s tests for multiple comparisons when an interaction was found to be significant. A value of *p* < 0.05 was considered statistically significant. All data were analysed using Prism 8 for Mac OS X, Version 8.0 (Graph Pad software).

## Results

### VAP-1 inhibition protects the BBB after LPS stimulus

**Fig. 1:**
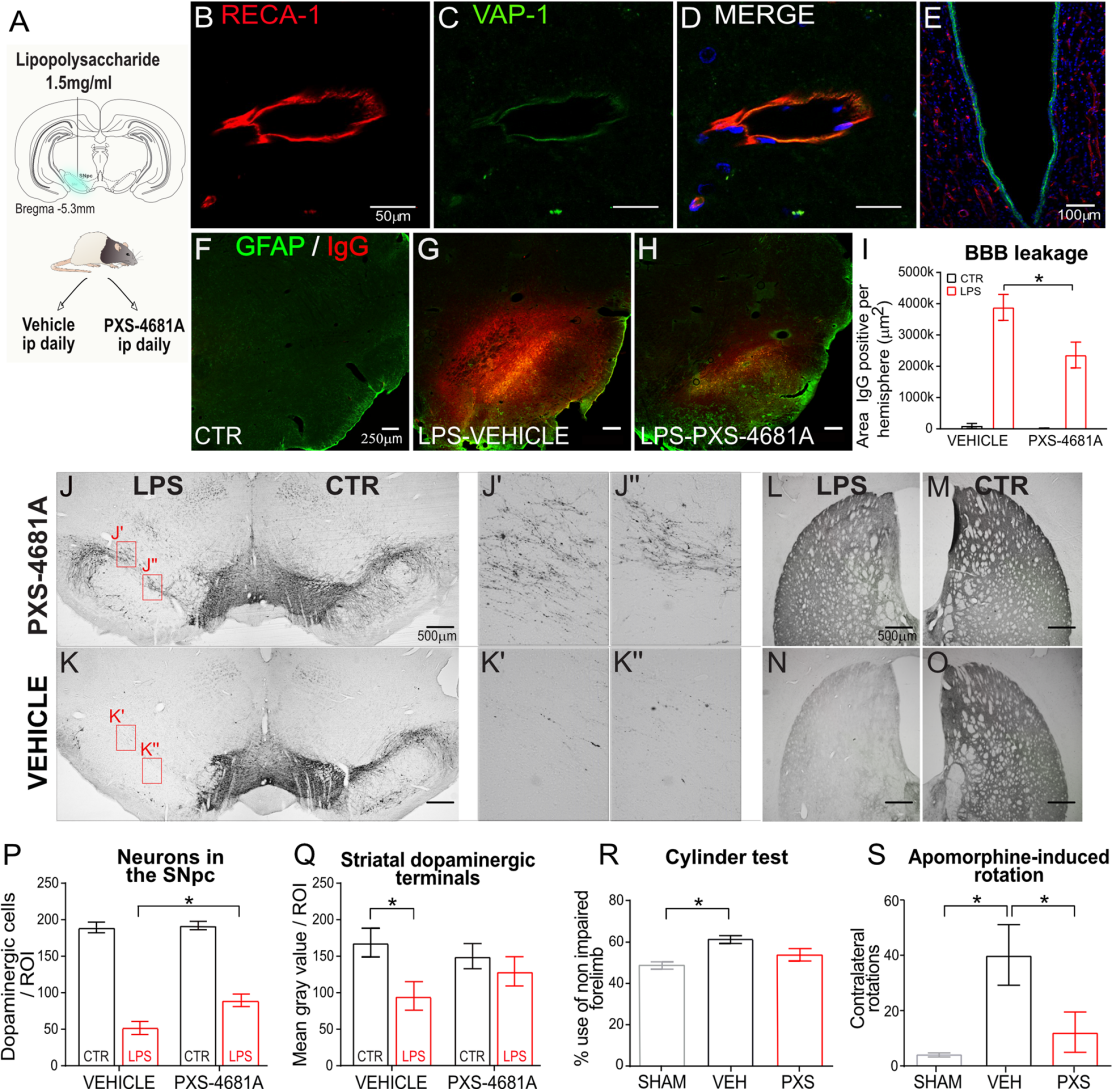
The inflammatory effect of LPS infusion into the SN and its attenuation by PXS-4681A.** A** Unilateral LPS injection and treatment regimen. **B**–**D** VAP-1 (green), RECA-1 (red) and Nissl (blue) in the SN after injection of LPS; **E** and on choroid plexus in the brain ventricle. **F–H** Rat-IgG (red) and GFAP (green) in control hemisphere **F**), LPS hemispheres–Vehicle **G** and LPS–PXS-4681A **H**. **I** Area IgG-positive in the LPS and CTR hemispheres in rats that received Vehicle or PXS-4681A ip. Bars represents mean ± SEM. LPS–Vehicle (*n* = 7), LPS–PXS-4681A (n = 5). **J**–**K** Coronal sections stained with TH in the SN after injection of LPS following PXS-4681A **J** or Vehicle **K** ip. **J’**, **J”**, **K’**, **K”** are enlargements of **J** and **K** images. **L**–**O** Representative photomicrographs of the density of DA terminals, identified as TH-positive, in the dorsal striatum of LPS hemisphere (left) and CTR hemisphere (right) from rats treated with PXS-4681A **L**, **M** or Vehicle **N**, **O**; **P** Quantification of TH-positive neurons in the SN. Values represent the mean ± SEM. LPS–Vehicle (*n* = 12) and LPS–PXS-4681A (*n* = 13). **Q** Quantification of TH-positive DA terminals in the dorsal striatum. Values represent the mean ± SEM. LPS–Vehicle (*n* = 12) and LPS–PXS-4681A (*n* = 13). **R** Asymmetry in forelimb use measured with cylinder test. LPS–Vehicle (*n* = 12), Sham–Vehicle (*n* = 6), LPS–PXS-4681A (*n* = 13). **S** Apomorphine-induced rotational behaviour, expressed as total number of contralateral rotations. Sham–Vehicle (*n *= 6), LPS–Vehicle (*n* = 6), LPS–PXS-4681A (*n* = 13). *p < 0.05

We have shown previously that ICV inflammation increases endothelial VAP-1 expression [36]. To confirm that local inflammation also induces measurable VAP-1 expression in the BBB, 24 h after local LPS injection into SN (Fig. 1A), the membrane-bound form of VAP-1 was found on the endothelial cells of the BBB but not in the parenchyma of the brain (Fig. 1B–D). We also observed strong VAP-1 expression on the choroid plexus in the brain ventricles, which has not previously been reported (Fig. 1E).

Because damage or leakage of the BBB can cause infiltration of immune cells that can lead to neuronal dysregulation and, ultimately, degeneration [11, 15, 48–50], we first assessed the role of VAP-1 in BBB damage induced by LPS infusion. Breakdown of the BBB was assessed by immunohistochemical detection of IgG extravasation [44]. Twenty-four hours after LPS there was massive infiltration of anti-rat IgG in the brain parenchyma (Fig. 1F–H) (LPS effect *F*_(1,10)_ = 91.38, *p* < 0.0001). VAP-1 enzyme activity was completely inhibited by ip injection of specific small-molecule inhibitor, PXS-4681A [35].

PXS-4681A prevented IgG infiltration suggesting that it protected BBB integrity: in contrast to the significant difference between the drug- and vehicle-treated LPS hemispheres (LPS–Vehicle *vs.* LPS–PXS-4681A), *p* = 0.014, there was no difference between drug- and vehicle-treated control hemispheres, producing a significant group x treatment interaction (repeated measures two-way ANOVA showed an effect of LPS, *F*_(1,10)_ = 91.38, *p* < 0.0001, no effect of PXS-4681A effect *F*_(1,10)_ = 4.160, but a significant interaction *F*_(1,10)_ = 5.045, *p* = 0.0485 (Fig. 1I).

### VAP-1 inhibition protects dopaminergic neurons after LPS stimulus

We next quantified DA cells 2 weeks after LPS infusion [38, 51], by manual count of TH-positive cells in the SN and TH-positive projections in the striatum, as the most commonly used marker for DA neurons.

TH in LPS–vehicle and PXS-4681A-treated animals differed significantly (Fig. 1J, K). The number of cells on the contralateral side was comparable in animals treated with PXS-4681A or vehicle indicating that LPS action was localized. In vehicle-treated rats, the number of TH-positive cells was 28% ± 4.79%, whereas in PXS-4681A-treated animals more cells survived, 46% ± 3.6% of the contralateral side; ANOVA showed a main effect of LPS, *F*_(1, 23)_ = 365.22, a main effect of PXS-4681A *F*_(1, 23)_ = 5.012, *p* = 0.0351 and an interaction between these variables, *F*_(1, 23)_ = 7.695, *p* = 0.011. Sidak’s multiple comparison revealed a significant effect in the LPS hemisphere, LPS–Vehicle *vs.* LPS–PXS-4681A, *p* = 0.003, but no difference in the control hemispheres (Fig. 1P).

DA neurons in the SN *pars compacta* project to the dorsal striatum and, therefore, the cell loss in SN caused a reduction of TH-positive staining in this projection area. This reduction was significantly diminished by PXS-4681A (Fig. 1L–O). The area of TH-positive staining contralateral to the LPS infusion did not differ whether the rat received PXS-4681A or vehicle, again confirming the localized action of LPS and the absence of an effect of the VAP-1 inhibitor on healthy tissue. On the ipsilateral side, vehicle-treated rats showed significantly lower TH-positive terminals (58.7% ± 7.51%) than PXS-4681A-treated animals (81.7 ± 4.89%) indicating a prominent protective effect of the drug. Repeated measures two-way ANOVA showed an effect of LPS, F_(1,23)_ = 25.06, *p* < 0.0001, no effect of PXS-4681A, *F*_(1,23)_ = 0.089, but a significant LPS x PXS-4681A interaction, *F*_(1,23)_ = 7.857, *p* = 0.01. Sidak’s multiple comparisons showed a significant difference between the LPS *vs.* control hemisphere in the Vehicle group (*p* < 0.0001) but no difference between hemispheres in PXS-4681A group (Fig. 1Q).

### VAP-1 inhibition induces motor recovery in LPS-lesioned rats

Unilateral DA neuron loss causes motor asymmetry and the effect of this loss was assessed by cylinder and apomorphine-induced rotation tests [52]. Cylinder test showed an increase in the use of the non-impaired forepaw, indicating a difference in level of dopamine between the two hemispheres, which was greater in the vehicle-treated LPS-lesioned group; i.e. they supported their bodies on the wall using the ipsilateral or non-impaired forelimbs 63.8% of the time. We first confirmed that the data satisfied both assumptions of parametric tests: test of normal distribution, Kolmogorov–Smirnov and Shapiro–Wilk, *p* > 0.05; and homogeneity of variance, Brown–Forsythe test and Bartlett’s test, *p* > 0.05. Subsequently, one-way ANOVA revealed a group difference *F*_(2,28)_ = 4.493, *p* = 0.0203. Sidak’s multiple comparisons then indicated Groups LPS–Vehicle significantly increased the ipsilateral forelimb use as compared to Sham group, *p* = 0.03. The group receiving PXS-4681A, on the other hand, showed a more even forepaw usage, reaching 55.4% use of the non-impaired forelimb, which was similar to the sham group, 51.3% (*p * > 0.05) (Fig. 1R).

Both cylinder test and apomorphine-induced rotation measure the asymmetric depletion of DA projections in the striatum [53], but they differ in the molecular mechanisms that induce stereotypical movements. Forelimb use in cylinder test is dependent on the DA activity in the striatum [54, 46] assessing the independent use of each forelimb in the context of a naturally occurring behaviour, whereas apomorphine stimulates D2 and D3 postsynaptic receptors, inducing abnormal contralateral rotations in hemiparkinsonian rats [55, 56]. In this specific experiment, we avoided to use amphetamine as its molecular structure is similar to the VAP-1 substrate, benzylamine, and that may cause interference with the rotational measurement.

Although we saw a reduction of DA neurons of about 70% in the SN and 40% in the striatum, apomorphine-induced turning behaviour revealed an overall low stereotypical rotation. However, as with the cylinder test, the apomorphine test showed a clear difference between groups, *F*_(2,14)_ = 6, *p* = 0.0093. Specifically, LPS-lesioned rats showed significantly higher rotation counts than sham rats (*p* = 0.010). The LPS–PXS-4681A group displayed low rotational activity, comparable to the values measured in the sham group (*p* > 0.05) and the LPS–PXS-4681A and LPS–Vehicle groups differed significantly (*p* = 0.0497) (Fig. 1S).

### VAP-1 inhibition reduces LPS-induced inflammation in the substantia nigra

**Fig. 2:**
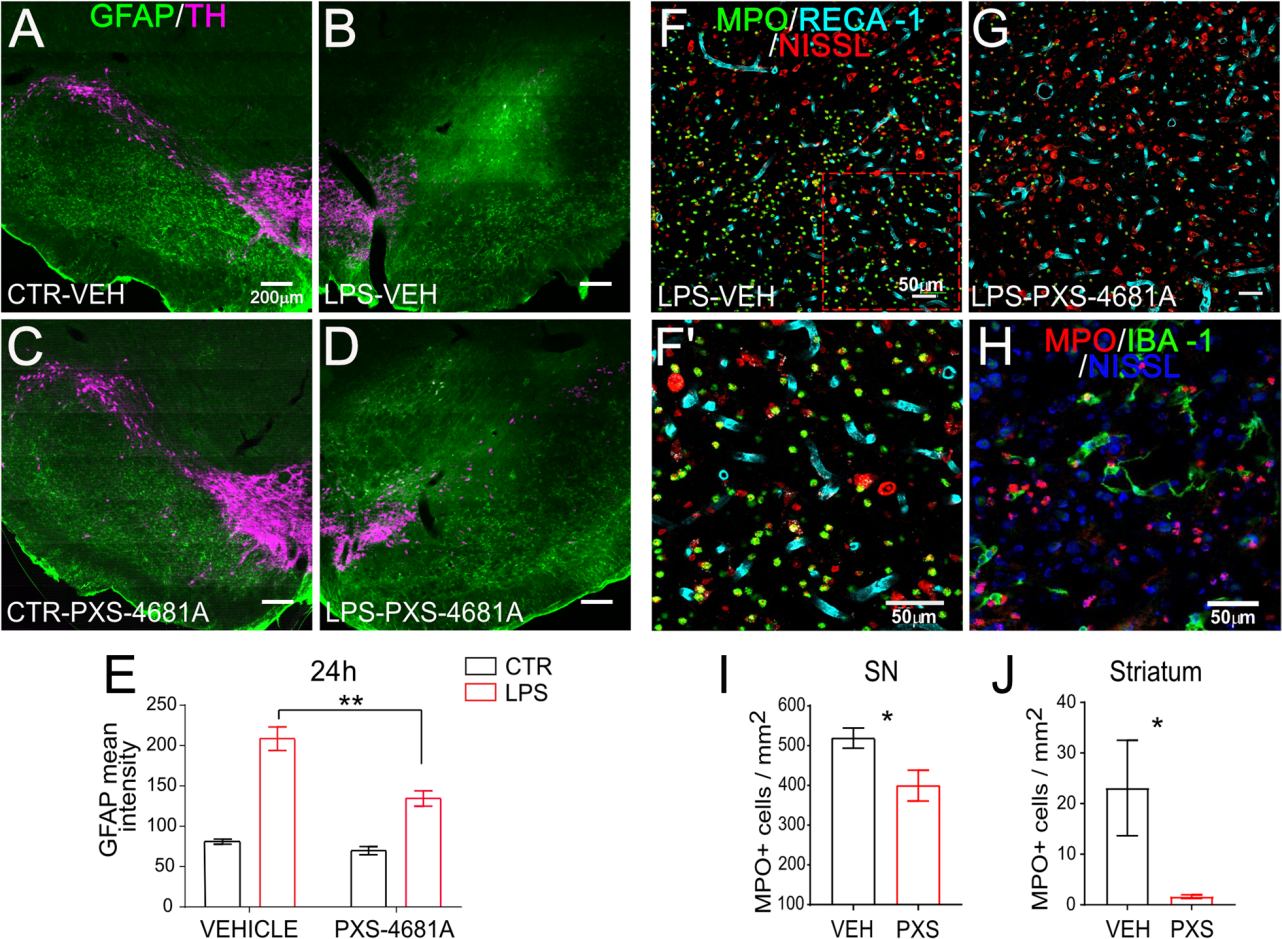
VAP-1 inhibition reduces the inflammatory response in the SN. **A**–**D** Coronal brain sections of SN immunostained for GFAP and TH revealed an increased GFAP intensity in the LPS hemispheres (**B** and **D**) as compared to CTR hemispheres (**A** and **C**). **E** Quantification of GFAP intensity in the SN at 24 h from LPS insult. Values represent the mean ± SEM. LPS–Vehicle (*n* = 7), LPS–PXS-4681A (n = 5). **F**, **G** Immunostaining for MPO, RECA-1-positive blood vessels and Nissl in the SN 24 h after LPS insult. **F’** Enlargement of **F**: MPO-positive cells mainly localized outside blood vessels. **H** Double immunostaining for MPO and Iba1 revealed a low percentage of colocalization, indicating that MPO-positive cells were mainly neutrophils. **I**, **J** Quantification of MPO-positive cells in the SN that received LPS **I** and in the ipsilateral striatum **J** at 24 h. Values represent the mean ± SEM. LPS–Vehicle (*n* = 7), LPS–PXS-4681A (*n* = 5). *p < 0.05; **p < 0.01

Following brain insult astrocytes react inducing astrogliosis [57, 58], which involves increased expression of the protein GFAP [59]. In normal conditions, GFAP staining in the SN is very low and mainly localized in the SN *pars reticulata* [60]. In our condition, GFAP immunofluorescence was increased in the SN after LPS infusion, corresponding to the area with high IgG signalling where the BBB had lost integrity (Fig. 1F–H); effect of LPS, *F*_(1,10)_ = 41.08, *p* < 0.0001 (Fig. 2A–D). VAP-1 inhibition decreased the LPS-induced response towards the levels in the control hemisphere (**Fig. ****2****E**) producing an effect of PXS-4681A, *F*_(1,10)_ = 6.155, *p* = 0.0325 and an LPS x PXS-4681A interaction, *F*_(1,10)_ = 5.545, *p* = 0.0403. Sidak’s multiple comparisons revealed a difference in the effect of PXS-4681A on GFAP intensity in the LPS treated hemispheres (LPS–Vehicle *vs.* LPS–PXS-4681A, *p* = 0.0055) but no effect in the control hemispheres.

Based on its properties as a VAP-1 inhibitor, we assessed the ability of PXS-4681A to control the leukocyte response in the SN. At 24-h post-lesion, there was a substantial accumulation of MPO-positive cells in the lesioned SN in both vehicle and PXS-4681A-treated rats (Fig. 2F, G), whereas this response was absent in the control hemispheres.

LPS challenge disrupts the BBB, and it is conceivable that the leakage of the vessel walls and the loss of tight junctions could cause unregulated entry of blood cells into the brain parenchyma. Most of the inflammatory cells infiltrating the SN were MPO-positive/Iba1-negative neutrophils (Fig. 2H), and as shown by co-labelling with RECA-1 antibody, MPO-positive cells were mostly localized within the tissue after BBB extravasation. Blocking VAP-1 was able to reduce the infiltration of leukocytes, as seen in the SN after PXS-4681A as compared to the LPS–Vehicle group (*t*-test, *p* = 0.012) (Fig. 2I).

### VAP-1 inhibition reduces the LPS-induced inflammatory response in the striatum

The intranigral injection of LPS was accompanied by an inflammatory response in the striatum, already visible at 24 h after LPS challenge; neutrophils were found extravasating from blood vessels specifically in the LPS hemisphere. PXS-4681A decreased the number of MPO-positive cells in the striatum (*t*-test, *p* = 0.049) (Fig. 2J).

**Fig. 3:**
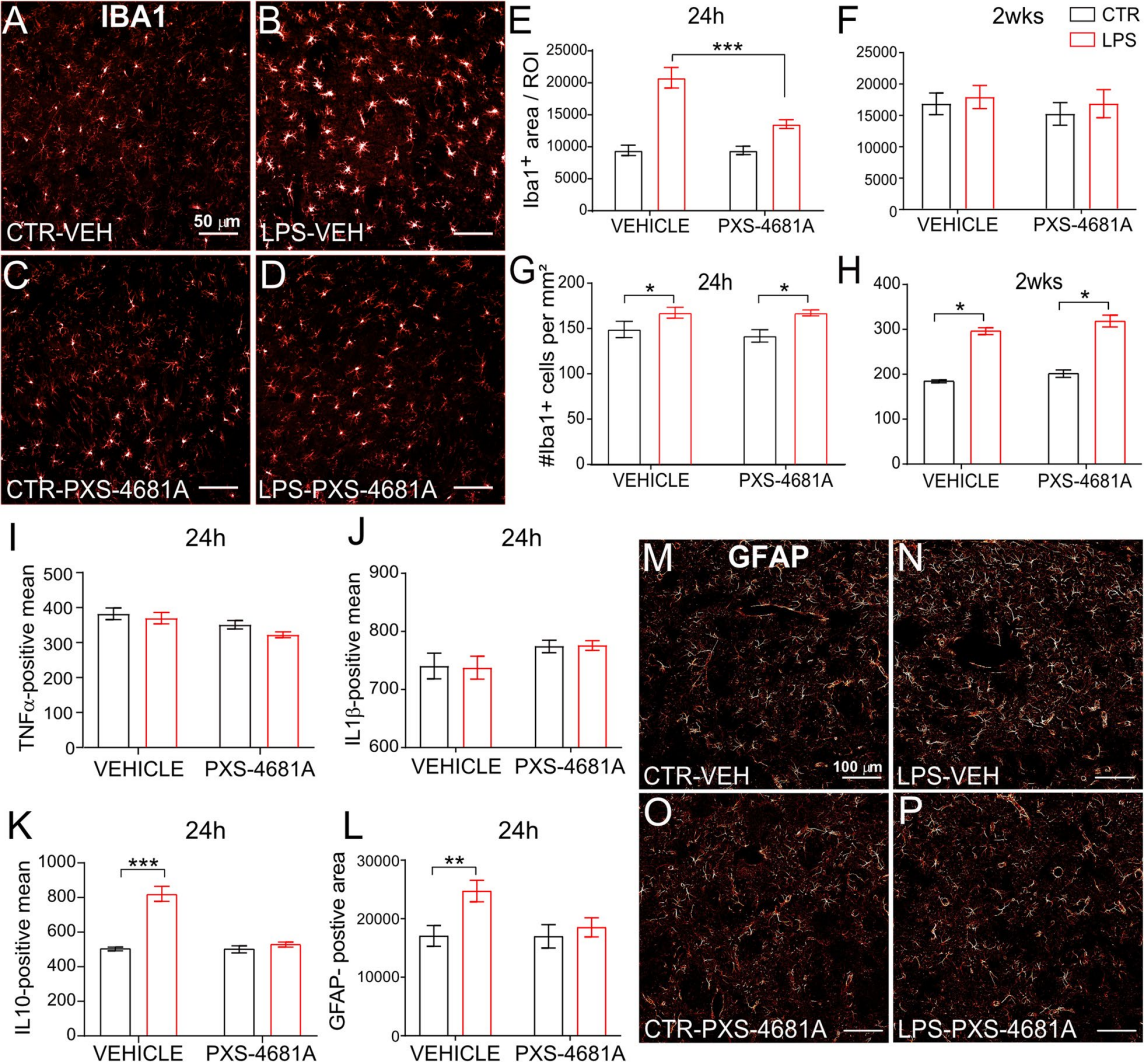
VAP-1 inhibition reduces the inflammatory response in the striatum.** A**–**D** Immunostaining for Iba1 cells in the striatum at 24 h. **E** Quantification of Iba1-positive area in the ipsilateral striatum (LPS) and contralateral striatum (CTR) at 24 h after injection of LPS in the SN. LPS–Vehicle (*n* = 7), LPS–PXS-4681A (*n* = 5). **F** Iba1-positive area in the striatum at 2 weeks. Values represent the mean ± SEM. LPS–Vehicle (*n* = 10); LPS–PXS4681A (*n* = 11). **G** Iba1-positive cell count in the striatum at 24 h after LPS insult. Values represent the mean ± SEM. **H** Iba1-positive cell count in the striatum at 2 weeks. Values represent the mean ± SEM. **I**–**K** Quantification of TNF-alpha, IL-1beta and IL-10 levels in the striatum at 24 h. Values represent the mean ± SEM. LPS–Vehicle (*n* = 7), LPS–PXS-4681A (*n* = 5). **L** Quantification of GFAP-positive area in the dorsal striatum 24 h. LPS–Vehicle (*n* = 7), LPS–PXS-4681A (*n* = 5). **M**–**P** Immunostaining for GFAP in the dorsal striatum. *p < 0.05; **p < 0.01, ***p < 0.001

In the striatum ipsilateral to the LPS infusion, the microglia cells, identified with Iba1 marker, showed increased cell counts and cellular hypertrophy, which resulted in an increased Iba1-positive area compared to the contralateral striatum, 24 h (LPS effect, *F*_(1,10)_ = 85.11) and 2 weeks (LPS effect, *F*_(1,19)_ = 6.038, *p* = 0.0238) after injection (Fig. 3A–D). This increased cell area was specifically attenuated at 24 h when the rats received PXS-4681A ip. (Interaction LPS x treatment, *F*_(1,10)_ = 16.55, *p* = 0.0028; Sidak’s multiple comparison showed a difference between the two LPS hemispheres at 24 h (*p* = 0.0005) but not between control hemispheres (*p* > 0.05) (Fig. 3E), nor at 2 weeks (Interaction, *F*_(1,10)_ = 0.2184) (Fig. 3F). Microglia numbers increased in the striatum at both time points, resisting PXS-4681A (Fig. 3G–H): two-way repeated measures ANOVA revealed an effect of LPS, *F*_(1,10)_ = 8.343, *p* = 0.0162; but neither an effect of PXS-4681A, *F*_(1,10)_ = 0.2836, nor a significant interaction, *F*_(1,10)_ = 0.2184, at 24 h timepoint; and an effect of LPS, *F*_(1,19)_ = 102.9, *p* < 0.0001; but neither an effect of PXS-4681A, *F*_(1,19)_ = 0.8767, nor a significant interaction, *F*_(1,19)_ = 0.1088, at 2 weeks after LPS.

Once activated, microglia undergo several key morphological changes including the uptake of MHC-II protein, and secretion of pro-inflammatory signalling molecules, detrimental to DA neurons [61, 62]. MHC-II expression, which plays a key role in CD4 + T cell activity [63, 64], increased at 2 weeks after LPS infusion (LPS effect *F*_(1,19)_ = 6.985, *p* = 0.0165) (Additional file 1: Fig. S1). VAP-1 inhibition did not affect this expression, suggesting that VAP-1 does not influence the CD4 response in the brain after LPS insult; PXS-4681A effect *F*_(1,19)_ = 0.0075, *p* > 0.05; Interaction *F*_(1,19)_ = 0.08489, *p* > 0.05.

We detected an overall low level of pro-inflammatory cytokines such as tumour necrosis alpha (TNF)-alpha and interleukin (IL)-1beta in the striatum. TNF-alpha was found to be decreased in the striatum in the presence of LPS (Fig. 3I), LPS effect *F*_(1,10)_ = 24.77, *p* = 0.001, whereas IL-1beta was unaffected (*F*_(1,10)_ = 0.006) (Fig. 3J). No main effect of PXS-4681A nor an interaction was detected for either cytokine. At the same time, IL-10 increased after LPS infusion in the ipsilateral hemisphere compared to the control hemisphere, LPS effect *F*_(1,10)_ = 22.92, *p* < 0.001. This effect was absent in the PXS-4681A group, PXS-4681A effect* F*_(1,10)_ = 18.15, *p* = 0.0017, producing a significant interaction *F*_(1,10)_ = 16.61, *p* = 0.0022: there was no difference between LPS and control hemispheres (significant difference between control and LPS hemisphere in the LPS–Vehicle group (*p* < 0.001), but not in the LPS–PXS-4681A group (*p* > 0.05)) (Fig. 3K).

As for microglia, striatal astrocytes also react to the LPS stimulus. Analysis of the GFAP-positive area in the dorsal striatum found an increase in response to the LPS infusion, *F*_(1,10)_ = 22.75, *p* = 0.008. Inhibition of VAP-1 decreased the LPS-induced response: there was  no effect of PXS-4681A, *F*_(1,10)_ = 1.468, but a significant LPS x PXS-4681A interaction was detected, *F*_(1,10)_ = 7.482, *p* = 0.021, indicating that there was a significant difference between control and LPS hemispheres in the Vehicle group (*p* = 0.0003) but not in the PXS-4681A group (Fig. 3L–P).

Severe nigrostriatal dopamine depletion and inflammatory response are both associated with postsynaptic changes on striatal spiny projection neuron morphology and modulation of striatal synaptic plasticity [65]. Two weeks after LPS challenge, we found LPS had no effect on the intensity of PSD-95-positive staining; LPS effect *F*_(1,23)_ = 0.3449, *p* > 0.05 (Additional file 1: Fig. S2A–D) but PXS-4681A was able to increase the intensity of PSD-95-positive puncta in both hemispheres as compared to Vehicle-treated rats (*F*_(1, 23)_ = 6.060, *p* = 0.0218) (Additional file 1: Fig. S2E). No Interaction *F*_(1,23)_ = 1.158, *p* > 0.05 was found and no effect in the number of PSD-95 puncta induced by either LPS or PXS-4681A was observed.

### VAP-1 inhibition restores habit learning induced by a loss of DA neurons in SN

**Fig. 4:**
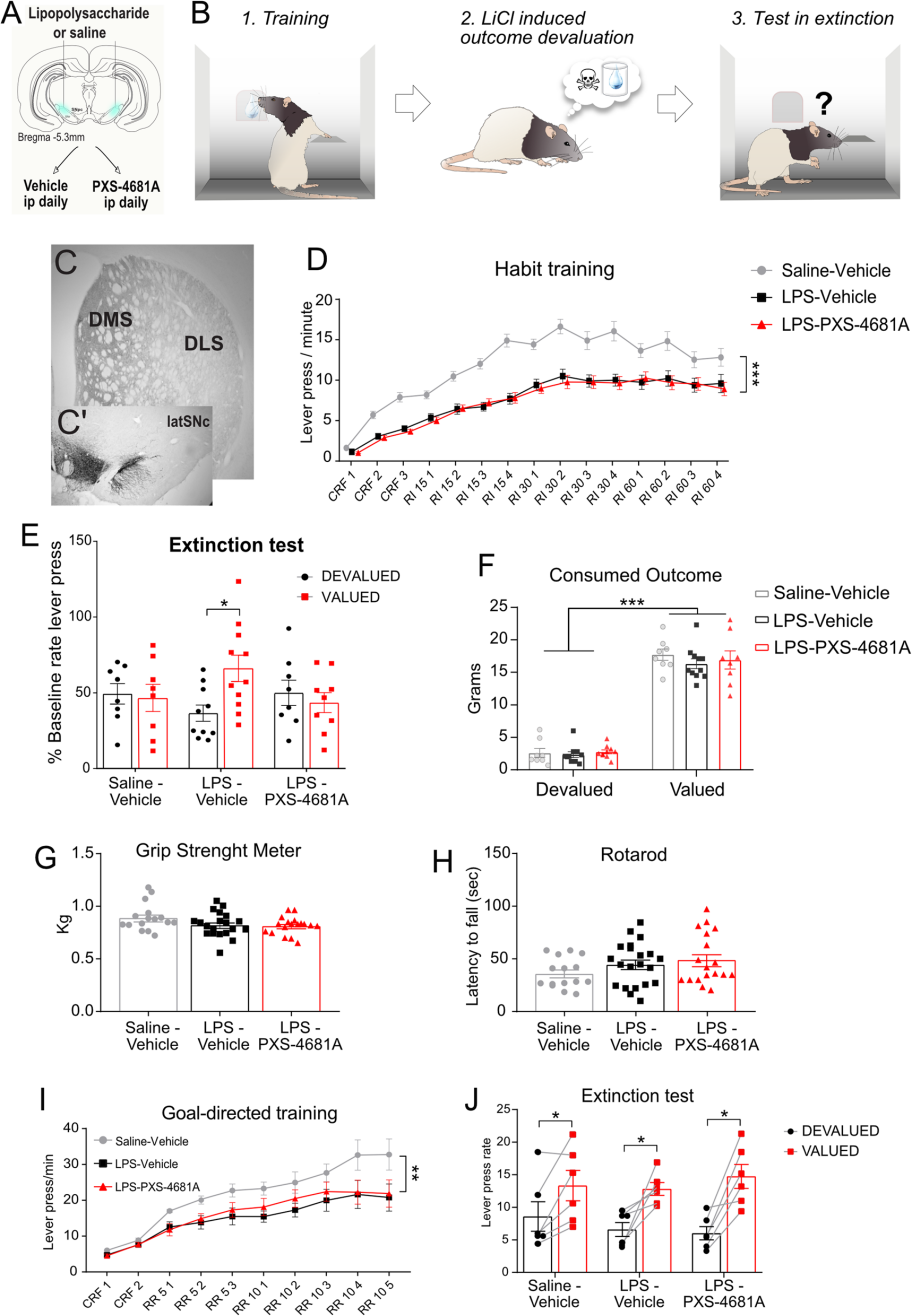
VAP-1 inhibition reverses the deficit in habitual motor learning.** A** LPS infusion. **B** Behavioural task. **C**–**C’** TH-immunoreactivity in the striatum **C** and in the SN **C’**) for the LPS-injected animals. **D** Lever pressing rate during training. Saline (*n* = 16), LPS–Vehicle (*n *= 21), LPS–PXS-4681A (*n* = 17). **E** Extinction test. The mean percentage as compared to baseline lever press rate for the devalued and the valued groups is shown. Saline–Vehicle devalued (*n* = 8), valued (*n* = 8); LPS–Vehicle devalued (*n* = 10), valued (*n* = 11); LPS–PXS-4681A devalued (*n* = 8), valued (*n* = 9). **F** Consumption test on day 3 of taste aversion devaluation. **G** Grip Strength Meter. **H** Rotarod performance. **I** Average of lever press rate of the two outcomes during training. Saline–Vehicle (*n* = 6); LPS–Vehicle (*n* = 6); LPS–PXS-4681A (*n* = 6). **J** Mean lever press rate during extinction test after specific satiety devaluation. Values represent the mean ± SEM. *p < 0.05; **p < 0.01, ***p < 0.001

In addition to motor impairment, loss of DA neurons in the lateral SN is associated with a loss of habit learning [66, 67] leading to a diminished ability to transfer routine activities to a less resource intensive process, increasing cognitive load and inducing cognitive interference. We sought to investigate the effect of VAP-1 inhibition on the impact of intranigral LPS on habit learning. LPS was injected bilaterally into the lateral SN (Fig. 4A), resulting in the disruption of the DA projection to the dorsolateral striatum (DLS) (Fig. 4C and C'). Goal-directed actions require the encoding of action–outcome (A–O) associations but, over the course of extended training, control over these actions shifts from a goal-directed process to a habitual process, the latter relying on the formation of stimulus–response (S–R) associations, rendering them insensitive to changes in outcome value, something that is usually demonstrated using an outcome devaluation test [68–70].

Two weeks after LPS was bilaterally infused into the lateral SN, food-restricted rats started 15 sessions of instrumental training (overtraining) to press a lever for a sucrose reward delivered on increasing random interval (RI) schedules (Fig. 4B and D). Groups were trained until they earned the same number of sucrose outcomes, so that they were exposed to the same number of reinforced lever presses. After training, the sucrose reward was devalued using LiCl-induced conditioned taste aversion for half the experimental group (using methods described in Lingawi and Balleine [71]) (devalued in Fig. 4E), whereas for the remainder it was not devalued (valued in Fig. 4E).

We anticipated that overtraining would induce habit formation and so reduce sensitivity to outcome devaluation in the Vehicle control group (i.e. valued = devalued) and this is what we found (Saline–Vehicle group in Fig. 4E). In contrast, bilateral infusion of LPS into the SN was predicted to produce a deficit in habit formation rendering lever pressing sensitive to outcome devaluation in the LPS–Vehicle group, which again was confirmed (LPS–Vehicle group in Fig. 4E). Importantly, VAP-1 inhibition by PXS-4681A rescued this deficit in habit learning, blocking the effects of LPS and generating the predicted insensitivity to outcome devaluation in the LPS–PXS-4681A group (Fig. 4E); ANOVA revealed a significant group x devaluation interaction *F*_(2,48)_ = 3.617, *p* = 0.0344; Sidak’s multiple comparisons test found a significant devaluation effect in the LPS–Vehicle group, *p* = 0.0143, but no devaluation effect in either the Saline–Vehicle and LPS–PXS-4681A groups, *p* > 0.05. The consumption of reinforcers during devaluation differed significantly between the devalued and valued groups (Fig. 4F), *F*_(1,48)_ = 491, but there were no differences among the devalued groups, which showed a similar taste aversion effect. A detailed analysis of performance during training indicated that the mean rate of lever pressing progressively increased for all groups (*F*_(4.681,238.7)_ = 124.2). However, LPS lesions produced an overall lower lever press rate compared to Saline–Vehicle (Group difference *F*_(2,51)_ = 20.67, Interaction *F*_(28,714)_ = 3.540, *p* > 0.001): Tukey’s multiple comparisons showed a significant difference between Saline and LPS–Vehicle groups (*p* < 0.0001), a significant difference between Saline and LPS–PXS-4681A (p < 0.0001) and no difference between LPS–Vehicle and LPS–PXS-4681A groups (Fig. 4D). As with the general failure to observe an effect on healthy tissue, there was, therefore, no clear evidence suggesting that PXS-4681A had an additional effect on performance to LPS, although this cannot be definitely confirmed. This decreased lever press performance in the LPS groups was not a result of impaired motor coordination or balance as neither rotarod nor grip strength meter tests showed differences between groups (Fig. 4G, H). Specifically, one-way ANOVA showed no group effect for rotarod, *F*_(2,51)_ = 1.726, *p* > 0.05, and no group effect for grip strength meter, *F*_(2,51)_ = 2.497, *p* > 0.05.

### LPS infusion into lateral SN and VAP-1 inhibition do not affect goal-directed action

It is conceivable that LPS blocked habit learning but that PXS-4681A enhanced goal-directed learning in a way unrelated to the LPS effect. To test this hypothesis, at the end of habit training and testing, rats were given sessions of training on two lever press actions with two different types of outcome (purified or grain pellets, counterbalanced), delivered on random ratio (RR) schedules to assess whether the groups differentially acquired new goal-directed actions. This training was followed by an outcome devaluation test which, following prior procedures—and to minimize any generalized effects of taste aversion treatment, used specific satiety-induced outcome devaluation [69]. All groups learned the new A–O contingencies, showing increased mean lever press rates over the course of training, *F*_(9,135)_ = 58.95, *p* < 0.0001, although the LPS lesion again reduced lever press rate compared to control (*F*_(2,15)_ = 3.597, *p* = 0.001; Fig. 4I). Nevertheless, if the new A–O associations were properly encoded, the animals should choose the action associated with the still valued outcome over the action associated with the now devalued outcome. Importantly, we found no effect of either LPS, PXS-4681A, or their interaction on the sensitivity of the lever press actions to outcome devaluation and a reliable devaluation effect was observed in each experimental group (Fig. 4J): two-way ANOVA showed an effect of devaluation, *F*_(1,15)_ = 68.74, *p* < 0.0001 but no Interaction between groups, *F*_(2,15)_ = 1.360. Sidak’s multiple comparisons showed a significant difference between devalued and valued actions in each group (*p* < 0.05). As such, these results suggest that the influence of LPS-induced dopamine neuron degeneration in the lateral SN was specific to habit learning and the rescue of habit learning by the VAP-1 inhibitor was not secondary to an off-target influence on goal-directed action.

## Discussion

VAP-1 has been extensively studied in the past for its role in mediating the infiltration of leukocytes in response to inflammatory stimuli [18]. In the brain, VAP-1 is expressed on the BBB endothelium; in inflammatory conditions, endothelial cells increase their expression of adhesion molecules, determining the type of leukocyte infiltrating the tissue [36]. Importantly, in the current series of experiments we found evidence of VAP-1 expression in SN capillaries and choroid plexus at the ventricles and we demonstrated that inhibiting VAP-1 not only targeted neutrophil extravasation, but also indirectly protected the BBB and attenuated both microglia and astrocyte responses at early time points. Furthermore, when the anti-inflammatory effect was lost at 2 weeks post-LPS, long after the neutrophil infiltration peak, the protective effect on neuronal function was still present, whereas there was no effect on microglia response, again suggesting that VAP-1 inhibition does more than simply affect the neutrophil response.

Our finding that VAP-1 inhibition protected DA neurons from degeneration and rescued behavioural function can be explained as a down-stream effect of an earlier modulation of neutrophil extravasation, as well as a consequence of the inhibition of SSAO enzymatic activity. In fact, VAP-1 is not only an adhesion protein; it also carries an enzymatic moiety that is widely diffused in the blood, with by-products such as ammonia, H_2_O_2_, formaldehyde and methylglyoxal increasing oxidative stress, vascular degeneration and protein unfolding [72–74]. Moreover, H_2_O_2_ has been implicated as an underlying factor in the initiation and progression of Parkinson’s disease. Increases in endogenous H_2_O_2_ in the dorsal striatum attenuated electrically evoked DA release and also decreased basal DA levels [75]. Indirectly, or at later stages [76], VAP-1 derived end-products also activate the endothelium, up-regulating E-selectin, chemokine CXCL8, vascular and intracellular adhesion molecule 1 (VCAM-1 and ICAM-1) resulting in increased lymphocyte adhesion [77]. It is plausible, therefore, that, even in the absence of a modulation of microglia response at 2 weeks, the inhibition of VAP-1 protects DA neurons by decreasing the production of ROS and vascular oxidative stress.

When injected into the SN, LPS induces an acute inflammatory response. The LPS-activated microglia and neutrophils release pro-inflammatory and neurotoxic factors such as IL-1, TNF-alpha, IL-6, NO and ROS that then cause neuronal damage [78, 79], suggesting that neuronal death is secondary to the inflammatory response [60]. Subsequently, the damaged neurons release neuromelanin and abnormal alpha-synuclein as injury signals to prompt reactive microgliosis [80–82]. The activation of microglia, via nicotinamide adenosine dinucleotide phosphate (NADPH) oxidase and toll-like receptor (TLR)-4, leads to further production of ROS and pro-inflammatory cytokines [83], inducing a self-amplifying cycle, which then results in a chronic inflammatory response and progressive DA neurodegeneration. In line with this hypothesis, an epidemiological study illustrated that the risk of developing Parkinson’s disease is significantly reduced by regular use of non-steroidal anti-inflammatory drugs such as ibuprofen [84].

It is not clear, however, whether intranigral LPS induces a chronic inflammatory response, mimicking the conditions under which microglia and neutrophils are activated in neurodegenerative diseases; nevertheless, it does recapitulate many features of Parkinson’s disease. First, DA neurons show a particular vulnerability to LPS-induced neutrophil infiltration [85, 86]; LPS is neurotoxic only in the presence of microglia [87, 88]; when injected into the striatum, LPS induces a progressive neuronal death over a 4-week period together with decreased motor performance on rotarod [89] and the accumulation of alpha-synuclein and ubiquitin [90]. These motor abnormalities can be improved by the administration of levodopa [89]. We suggest, therefore, that LPS can provide a useful tool to investigate the effect of the inflammatory process on DA neurons and to assess new anti-inflammatory drugs, particularly in conditions where an acute inflammatory response and neutrophil infiltration are observed. However, with intranigral LPS injections, the progression of the DA loss is relatively fast, limiting the study to a neuroprotective approach.

As with other toxins that are used to induce DA neuronal death, stereotaxic injection of LPS reduces the activity of mitochondrial complex I of the electron transport chain in both SN and striatum [90], similar to what is observed in Parkinson’s patients [91]. The mechanism is associated with the upregulation of NADPH oxidase, essential to the production of superoxide [90, 92], and detrimental to DA neurons [92].

LPS direct injection also downregulates superoxide dismutase and glutathione peroxidase, two important antioxidant enzymes [93], whose absence can exacerbate neuronal dysfunction and death [92]. Currently, there is no clear evidence that SSAO/VAP-1 inhibition can directly modulate these enzymes; however, aminoguanidine, an SSAO inhibitor, has been shown to reduce nitric oxide synthase [94, 95], which normally leads to the production of superoxide at the expense of NADPH. In addition, augmented SSAO immunoreactivity appears to be associated with elevated Cu/Zn superoxide dismutase 1 expression in blood vessels of Alzheimer’s disease brains [26], possibly to protect cells from the SSAO-released superoxide. Indeed, the plasma of patients with sporadic Alzheimer’s disease at moderate–severe and severe stages, has shown increased activity from circulating SSAO/VAP-1, compared to healthy controls [96]. These results highlight the importance of the circulating enzyme, which possibly derives from the shedding of the membrane-bound VAP-1, and results in increased ROS production not only at the site of inflammation, but also systemically, which can further contribute to oxidative stress and vascular damage. We are, therefore, supportive of the hypothesis that the neuronal protection provided by the inhibition of SSAO/VAP-1 in the current study comes from both a local decrease in the infiltration of neutrophils and ROS production as well as from a more systemic effect in the blood stream, which prevents vascular dysfunction and downstream effects. However, the specific mechanism of this latter effect must await future experiments.

When injected into the SN, LPS binds to the TLR-4 expressed on endothelial cells and on microglia but not on astrocytes [97]. Within 24 h from the injection, the microglia population disappears [85], leaving the endothelium to drive the inflammatory cascade, which allows the infiltration of neutrophils and primes the astrocytic response in the SN. An interaction between activated endothelium and astrocytes is also evident in models of Parkinson’s disease [58] and in Alzheimer's disease patients, where there is an increase of reactive astrocytes in vessels with a higher expression of VAP-1 [25].

In the striatum LPS resulted in fewer neutrophils infiltrating and adhering to vessels compared to SN and both astrocytes and microglia cells increased their signature markers at 24 h post-LPS. The synchronous response of these two cell types suggests they may be acting interdependently and, as observed in LPS systemic models and Alzheimer's disease [97–99], LPS exposure promotes the M1 microglia phenotype that consequently elicits the shift to an A1 phenotype in astrocytes. However, at 24 h after LPS neither IL-1beta nor TNF-alpha were significantly increased in the striatum, whereas they have been reported to increase in the SN [100]. It is possible that in the striatum pro-inflammatory cytokines peak earlier [60] or for a shorter period and that at 24 h only resolving cytokines, such as IL-10, are released [101]. IL-10, generally known as an anti-inflammatory cytokine, is increased in the striatum. This is not surprising as it peaks later than pro-inflammatory cytokines. However, recent research has established that IL-10 can be released by neutrophils [102] as a restorative mechanism after interaction with LPS-stimulated Treg cells and that IL-10 promotes apoptosis in monocytes/macrophages [103] and in neutrophils [104].

The spiny projecting neuron is the target of the dopamine innervation of the striatum, and comprises more than 90% of striatal neurons. In Parkinson patient’s, striatal DA denervation correlates with striatal spine loss on spiny projecting neurons and remodelling of axospinous glutamatergic synapses. Loss of striatal spines is an early event that does not correlate with the severity of motor symptoms, suggesting that might be a compensating adaptation for the loss of striatal DA innervation in the early stages of the disease [105]. Both post-mortem idiopathic Parkinson’s patients and animal models using 6-OHDA and MPTP, show loss of dendritic spines induced by striatal dopamine depletion [106–109]. We quantified postsynaptic puncta of medium spiny neurons in the dorsal striatum using PSD-95-positive staining and observed that the LPS lesion did not affect the intensity of this staining. PSD-95, important for restoring physiological synaptic functioning, is upregulated by the VAP-1 inhibitor and its restoration is possibly important to ameliorate brain function. However, more investigation on this specific aspect is required.

Several new anti-inflammatory drugs are now in clinical trials for Parkinson’s disease [110, 111]. However, due to the complexity of the disease and the interactions of different mechanisms, design of a successful intervention to protect the nigrostriatal pathway will likely require a multi-target approach initiated during the earliest stages of disease. This is particularly important because inflammation is considered an early event in the progression of Parkinson’s disease and other neurodegenerative diseases and early diagnosis is key to preventing DA loss. In this regard, specific analysis of inflammatory markers has been suggested as diagnostic biomarkers [111].

### Functional deficits associated with the loss of DA neurons

Less attention has been given to non-motor symptoms in LPS animal models of Parkinson’s disease and, considering that the onset of non-motor symptoms precedes the onset of motor symptoms, it is important to understand non-motor aspects. In the current study, we found that a specific effect of LPS infusion into lateral SN was a loss of habit learning. Blockade of the VAP-1 receptor after LPS infusion reduced the neuropathological hallmarks of the insult and rescued the learning deficit caused by the loss of DA terminals in the DLS. Dopamine loss in the striatum is not only associated with motor impairments, but also with cognitive decline. The loss of DA neurons in Parkinson’s disease patients starts in the lateral part of the SN and worsens as the condition endures [67]. The lateral SN projects to the sensorimotor putamen [112], which is implicated in decision-making, particularly in the control of habitual actions [113, 114]. When making a choice amongst several alternatives, we usually deliberate over the consequences of actions and then select an action based on its value. This process is called goal-directed decision-making [115]. Under invariant conditions, however, actions can become more automatic as they come under habitual control*.* In Parkinson’s disease patients, learning and performance processes subserving habitual action control are impaired, forcing patients to maintain cognitive control of even very commonly performed actions, resulting in deficits in executing fast, automatic actions.

We found that bilateral LPS injections into the lateral SN disrupted the influence of habit learning on instrumental performance, and that treatment with PXS-4681A reversed this deficit. This is relevant because, despite producing only a partial ~ 20% protection from neuronal loss and the ability to control the inflammatory response only in the early stages after the insult, PXS-4681A potently rescued this important behavioural function. Furthermore, PXS-4681A was neuroprotective without affecting the function of other brain areas; we found, for example, that goal-directed actions—which are mediated by the dorsomedial striatum [116]—were unaffected by its administration. We speculate that, by decreasing the inflammatory response in the SN and striatum at early time points, PXS-4681A rescued DA loss and decreased the neuronal stress induced by the inflammatory response. This also resulted in the restoration of cognitive and motor functions affected by the loss of dopamine. The results of this study suggest, therefore, that VAP-1 inhibition may be a relevant target for protecting DA neurons from acute inflammatory stimuli and subsequent cell death and indicate that VAP-1 inhibition is likely to be particularly effective in reducing the effects of inflammatory responses in the SN in situations where the neutrophilic component is of relevance.

## Supplementary Information

**Additional file 1. Figure S1.** MHC-II expression in striatal microglia. **Figure S2. **VAP-1 inhibition increases postsynaptic protein in the striatum.

## Data Availability

The datasets used and/or analysed during the current study are available from the corresponding author on reasonable request.

## Abbreviations

BBB: Blood–brain barrier
CRF: Continuous reinforcement
DA: Dopaminergic
DAB: 3,3’-Diaminobenzidine
DLS: Dorsolateral striatum
GFAP: Glial fibrillary acidic protein
Iba1: Ionized calcium binding adaptor molecule 1
ICV: Intracerebroventricular
IL: Interleukin
IP: Intraperitoneal
LPS: Lipopolysaccharide
MHC: Major histocompatibility complex
MPO: Myeloperoxidase
NADPH: Nicotinamide adenosine dinucleotide phosphate
PBS: Phosphate buffer saline
PFA: Paraformaldehyde
PSD-95: Postsynaptic density protein 95
RECA: Rat endothelial cell antibody
RI: Random interval
RR: Random ratio
SN: Substantia nigra
SSAO: Semicarbazide-sensitive amine oxidase
TH: Tyrosine hydroxylase
TNF: Tumour necrosis factor
VAP-1: Vascular adhesion protein 1
