# Supplementary material for: Inhibition of vascular adhesion protein 1 protects dopamine neurons from the effects of acute inflammation and restores habit learning in the striatum
Source: J Neuroinflammation. 2021 Oct 15;18:233. doi: 10.1186/s12974-021-02288-8 (PMC8520223; doi:10.1186/s12974-021-02288-8)
Supplement: Supplementary file 1 — Additional file 1. Figure S1. MHC-II expression in striatal microglia. Figure S2. VAP-1 inhibition increases postsynaptic protein in the striatum. [file 12974_2021_2288_MOESM1_ESM.docx]

**Inhibition of vascular adhesion protein 1 protects dopamine neurons**

**from the effects of acute inflammation and restores**

**habit learning in the striatum**

***Additional file 1***

*

*

**Figure S1. MHCII expression in striatal microglia.**

A) Quantification of MHC-II expression in Iba1-positive cells in the striatum at 2 weeks after LPS insult in the SN.

**
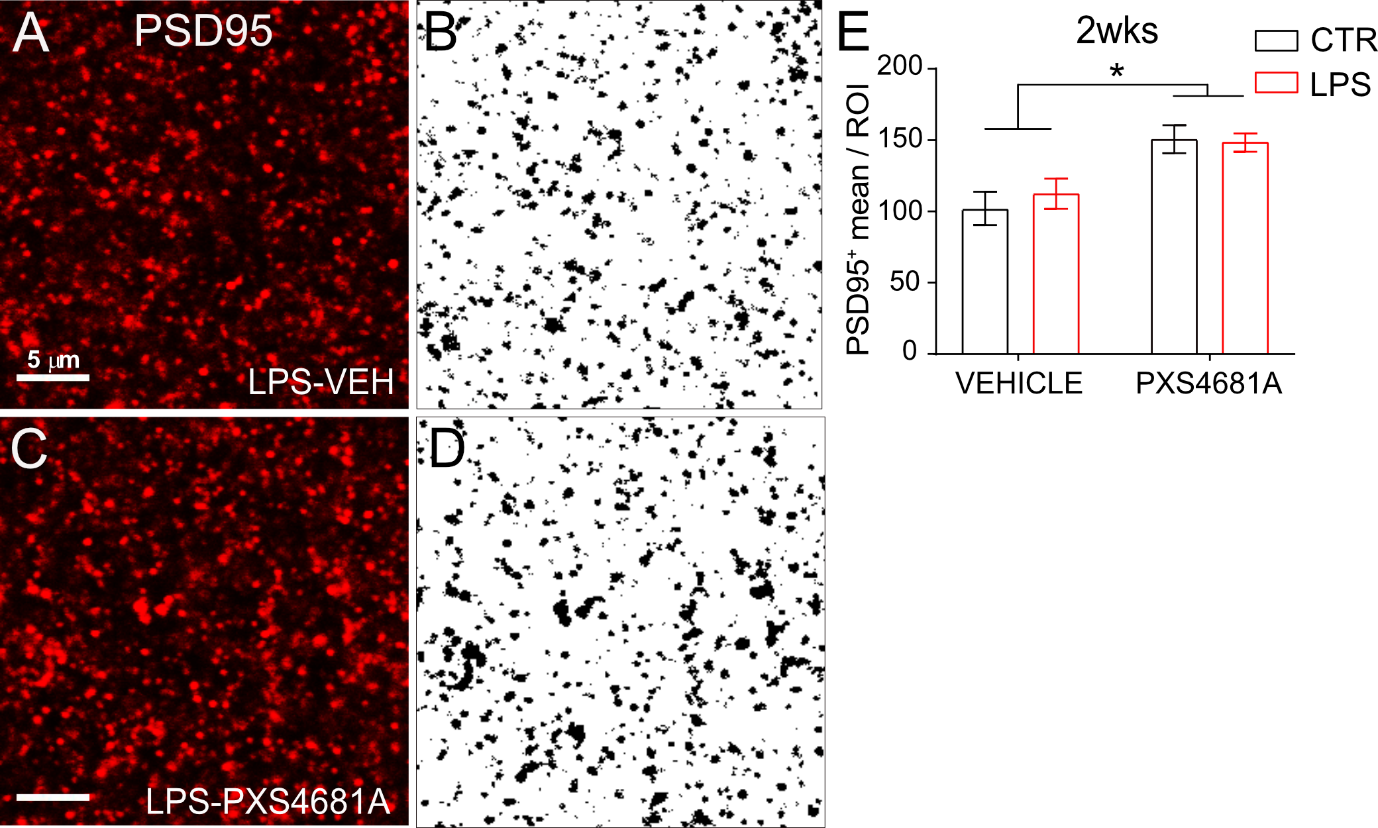
**

**Figure S2. VAP-1 inhibition increases postsynaptic protein in the striatum.**

**A-D**) Immunostaining for PSD-95 in the dorsal striatum and example of quantification as positive area using Image J.

**E**) Quantification of immunostaining for PSD-95 in the dorsal striatum 2 weeks after LPS insult in the SN. LPS-Vehicle (n = 12); LPS-PXS-4681A (n = 13).
